# Intra-abdominal pressure and residual renal function decline in peritoneal dialysis: a threshold-based investigation

**DOI:** 10.1080/0886022X.2024.2312535

**Published:** 2024-02-06

**Authors:** Jingjing Zhang, Lei Song, Zhongwei Ma, Lina Sun, Xiaoqing Wang, Duanyan Liu, Feng Huang, Yulin Man

**Affiliations:** a Graduate School of Jinzhou Medical University, Jinzhou, PR China; b Department of Nephrology, Linyi People’s Hospital, Linyi, PR China

**Keywords:** Peritoneal dialysis, intra-abdominal pressure, residual renal function

## Abstract

**Background:**

The potential impact of elevated intra-abdominal pressure (IAP) on residual renal function (RRF) has not been determined. The objective of this study was to investigate the relationship between IAP and the rate of RRF decline in newly initiated peritoneal dialysis (PD) patients, and to identify the optimal IAP threshold value for delaying the deterioration of RRF.

**Methods:**

A cohort of 62 newly initiated PD patients who completed both 6- and 12-month follow-up evaluations was obtained using the Durand method. A logistic regression model was used to identify variables associated with a rapid decline in RRF. Receiver operating characteristic (ROC) curves were generated to determine the optimal threshold value. Another retrospective cohort analysis was performed to validate the identified critical value.

**Results:**

For each 1 cmH_2_O increase in IAP, the risk of a rapid decline in the RRF increased by a factor of 1.679. Subsequent analysis revealed that patients in the high IAP group had more significant decreases in residual renal estimated glomerular filtration rate (eGFR) (*Z* = −3.694, *p* < 0.001) and urine volume (*Z* = −3.121, *p* < 0.001) than did those in the non-high IAP group. Furthermore, an IAP ≥15.65 cmH_2_O was a robust discriminator for the prediction of the rate of RRF decline.

**Conclusion:**

Patients in the high IAP group experienced a more rapid decline in RRF. Additionally, an optimal critical pressure of 15.65 cmH_2_O was identified for predicting the rate of RRF decline. IAP, as one of the factors contributing to the rapid decline in RRF in the first year of PD, should be given due attention.

## Introduction

Approximately 11% of the global population of dialysis patients are receiving peritoneal dialysis (PD), a modality for renal replacement therapy [1]. This method employs the peritoneum as a semipermeable membrane, infusing dialysate into the peritoneal cavity. It establishes a transmembrane pressure gradient between the blood and dialysate, facilitating the removal of water and solutes [2]. Notably, due to inherent patient-specific variations, certain individuals manifest elevated peritoneal pressure subsequent to the routine infusion of 2 L of dialysate.

According to the World Society of the Abdominal Compartment Syndrome (WSACS), intra-abdominal pressure (IAP) is defined as persistent IAP ≥ 12 mmHg and abdominal septal compartment syndrome is defined as persistent IAP > 20 mmHg (1 mmHg = 1.36 cmH_2_O), with or without organ dysfunction or failure [3]. Animal experiments have consistently demonstrated that elevated IAP levels lead to an increase in renal vascular resistance, resulting in reduced renal blood perfusion, a diminished glomerular filtration rate (GFR), and subsequent renal function impairment, which may include oliguria or anuria [4–7].

In the context of PD patients, a considerable concern revolves around the potential impact of elevated IAP on the rate of residual renal function (RRF) decline. The RRF represents the residual capacity of native kidneys to excrete waste products and maintain electrolyte balance, and constitutes a pivotal asset in the management of end-stage kidney disease (ESKD)[8]. In dialysis patients, maintaining RRF is extremely important for improving quality of life and reducing mortality [9,10]. Notably, routine measurements of the IAP in patients with PD are currently unavailable from standard nephrology practice.

In this study, we conducted a comprehensive investigation of a cohort of patients with newly initiated PD to elucidate the impact of elevated IAP on RRF decline and, importantly, to determine an optimal threshold for the IAP that could help delay the deterioration of RRF. Understanding this relationship is essential for clinicians and health care providers in their efforts to optimize PD therapy and patient outcomes.

## Materials and methods

### Patient enrollment

This prospective cohort study was registered in the Chinese Clinical Trial Registry (registration number: ChiCTR2300067989). Between July 2021 and June 2022, we recruited a cohort comprising 62 patients with newly initiated PD. These patients had previously undergone PD catheter insertion and subsequently completed the requisite 6- and 12-month follow-up assessments at the PD Center located within Linyi People’s Hospital. All 62 patients underwent continuous ambulatory PD (CAPD). PD catheter insertion procedures were executed using open abdominal surgery or laparoscopic techniques.

The inclusion criteria for patients were as follows: (1) aged older than 18 years; (2) at 6- and 12-months postsurgery, 24-h urine samples were collected; and (3) at the 6-month time point, RRF defined as a 24-h urine volume exceeding 100 mL was maintained. The exclusion criteria were as follows: (1) peritonitis following PD catheter insertion; (2) peritonitis, heart failure (NYHA class 2 and above), and liver failure during the follow-up period as well as treatment involving both PD and hemodialysis (HD); (3) transition to HD or kidney transplantation; (4) noncompletion of follow-up. This study was approved by the Ethics Committee of Linyi People’s Hospital under protocol number YX200526.

### Data collection

The data were derived from eligible patient cases. Basic demographic characteristics, including age, sex, height, weight, body mass index (BMI), and body surface area (BSA), were recorded. Additionally, the etiology of ESKD, comorbidities, medications prescribed during follow-up, and PD prescriptions were recorded. Vital signs such as systolic and diastolic blood pressure, and heart rate, were documented. The relevant laboratory data consisted of baseline estimated GFR (eGFR), serum ALB concentration, blood urea nitrogen concentration, blood creatinine concentration, 24-h urinary urea nitrogen concentration, 24-h urinary creatinine concentration, and other pertinent parameters. Furthermore, specific dialysis-related information, such as 24-h urine volume, 24-h ultrafiltration volume, residual renal eGFR, and results from the peritoneal equilibrium test (PET), was meticulously obtained.

### Calculation of RRF

The baseline eGFR before PD catheter insertion was determined using the chronic kidney disease epidemiology collaboration (CKD-EPI) formula as recommended by the 2012 Kidney Disease: Improving Global Outcomes (KDIGO) guidelines [11]. The RRF during follow-up was calculated as the arithmetic mean of 24-h urea nitrogen and creatinine clearance [12], measured every 6 months. Before the collection period, patients emptied their bladder by spontaneous voiding, and all urine passed thereafter was collected (including a final void at the end of the 24-h period). Within the observation period, patients underwent two inpatient 24-h urine collections at the 6th and 12th months. The monthly rate of RRF decline was calculated as the arithmetic mean of the decline in RRF. The primary efficacy event of the study was whether the monthly rate of RRF decline for each patient at the end of the observation period (12th month) reached the average monthly decline rate of the overall sample.

### Peritoneal equilibrium test

After six months of CAPD, PET was conducted using Twardowski’s method [13]. In preparation for PET, 2.5% of the peritoneal fluid was allowed to settle in the peritoneum for 8–12 h the day before the test and subsequently drained the following morning. Following this, 2 L of 2.5% peritoneal fluid was instilled into the peritoneal cavity, and the instillation time was recorded as 0 h. Peritoneal fluid creatinine and serum creatinine samples were collected at 0, 2, and 4 h. The peritoneal transport characteristics were assessed by calculating the dialysate-to-plasma ratios of creatinine at four hours (D/P4).

### Measurement of the IAP

Durand’s method [14] was used to measure IAP in patients who were receiving maintenance PD treatment at 6 and 12 months. Following bladder emptying, patients were assumed to be in a supine position, using the axillary midline as a reference point. The PD double-bag system was connected. The dialysate residing in the abdomen was drained into the waste fluid bag and subsequently clamped after complete drainage. The inlet pipeline was opened to introduce the dialysate into the peritoneal cavity. The short external tube remained open throughout the process. Sterilization of the dosing port of the fluid entry bag with iodine vapor was performed, and the introduction cannula needle was connected to atmospheric pressure to ensure the absence of reverse pressure in the pipeline system. After the liquid column height was allowed to stabilize for 1 min, dialysate height measurements in the fluid-entry pipeline were recorded at the end of expiration and inhalation, during two repetitions, with the average value considered for analysis.

IAP measurements of patients with PD were conducted at both 6- and 12-month intervals. The calculated average of these two measurements was used for further analysis. Subsequently, patients were categorized into distinct groups based on their IAP values, specifically, the nonhigh IAP group (IAP < 15.65 cmH_2_O) and the high IAP group (IAP ≥ 15.65 cmH_2_O), depending on whether their IAP surpassed the threshold of 15.65 cmH_2_O.

### Statistical analysis

All the statistical analyses were performed using SPSS software version 26.0 (SPSS Inc., Chicago, IL). Continuous variables are presented as the mean ± standard deviation. The independent samples t-test was used for normally distributed data, while the Mann–Whitney U test was used for nonnormally distributed data. Count data are expressed as frequencies and percentages and were analyzed using the Chi-square test. The multiple Kruskal–Wallis test was used to compare residual kidney eGFR at the three-time points. For peritoneal transport characteristics, baseline data were used for analysis. For other time-dependent parameters, including blood pressure, heart rate, and serum chemistry, average values during follow-up were used for analysis. Logistic regression analysis was performed to identify statistically significant risk factors associated with a rapid decrease in RRF. Starting with a univariate analysis, variables demonstrating significance in the univariate analysis (*p* < 0.05) were incorporated into a multivariate analysis to determine whether they were independently predictive of RRF decline. Spearman’s correlation analysis was used to assess the relationship between IAP and changes in RRF. Receiver operating characteristic (ROC) curves were constructed to assess the diagnostic utility of the IAP for predicting the rate of RRF decline, and the Youden index was computed to identify the optimal critical IAP value. The discriminative ability of the optimal cutoff value was evaluated using area under curve (AUC). *p* < 0.05 was considered indicative of statistical significance.

## Results

### General information

**Figure 1.**
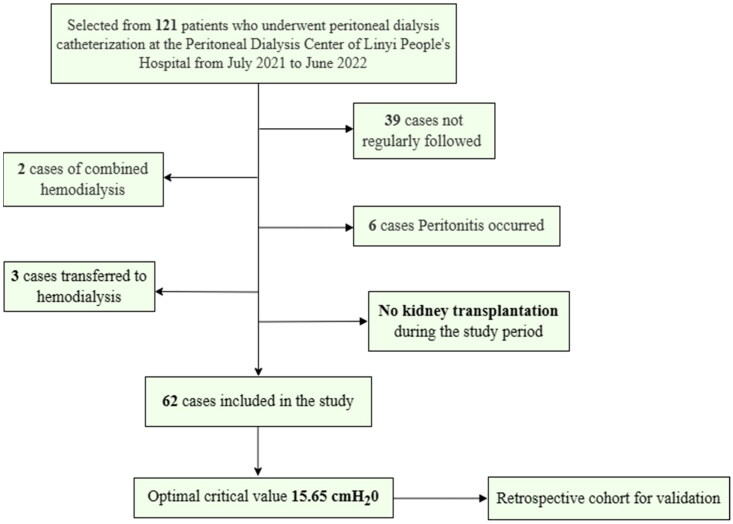
Flow diagram of study inclusion.

The subject enrollment process for the prospective cohort is shown in Figure 1. Among the initial 121 screened patients, 62 met the inclusion criteria and completed follow-up. Two patients took immunosuppressants because the primary disease was systemic lupus erythematosus (SLE), while six patients were taking nonsteroidal anti-inflammatory drugs (NSAIDs) for cardiovascular disease. At the end of the follow-up period, no patients had died. Some patients had undergone preceding acute HD before changing to long-term PD therapy. Among these, 37 patients underwent open abdominal surgery, while 25 patients underwent surgery *via* a laparoscopic approach. The mean age of the included patients was 50.53 ± 13.29 years, and most were female (*n* = 39). The causes of end-stage renal disease included hypertension (*n* = 20), chronic glomerulonephritis (*n* = 18), diabetes mellitus (*n* = 17), obstructive nephropathy (*n* = 2), hepatitis B nephropathy (*n* = 1), SLE (*n* = 2), and polycystic kidney disease (*n* = 2). All patients had a dialysate glucose concentration of 1.5%. There were three types of PD prescriptions for both groups as follows: prescription 1 involved filling the intraperitoneal cavity with 2000 mL of a 1.5% dextrose solution, exchanged twice daily, with a dwell time of 4 h each; prescription 2 entailed filling the intraperitoneal cavity with 2000 mL of a 1.5% dextrose solution, exchanged three times daily, with a dwell time of 4 h each; prescription 3 involved filling the intraperitoneal cavity with 2000 mL of a 1.5% dextrose solution, exchanged four times daily, with a dwell time of 4 h each. The average D/P4 for all patients was 0.66 ± 0.13, and the majority of patients exhibited high-average or low-average peritoneal transport characteristics. The baseline characteristics are presented in Table 1.

### Changes in the residual renal eGFR, urine volume, and ultrafiltration volume in patients with PD

After 6 months of treatment, the residual renal eGFR in the 62 patients decreased from 5.2 mL/min/1.73 m^2^ at baseline to 3.41 mL/min/1.73 m^2^, and further to 2.75 mL/min/1.73 m^2^ at 12 months, exhibiting a significant decreasing trend (*H* = 37.445, *p* < 0.001). The decline was substantial, with a reduction of 40.94% at 6 months and 59.06% at 12 months from baseline, both of which were statistically significant. The 24-h urine volume decreased from 1117 mL at 6 months to 894 mL at 12 months, with a statistically significant difference between the two time points (Z = −2.113, *p <* 0.05). Additionally, the IAP measured at 12 months (Z = −2.121, *p <* 0.05) was significantly lower than that measured at 6 months. However, no statistically significant differences were observed in the ultrafiltration rate (*p* > 0.05) between the 6- and 12-month follow-up data (Table 2).

**Table 2.** Changes in residual renal function in patients with newly initiated PD.

Variables		H/t/z	*p* Value
Residual eGFR (mL/min/1.73 m^2^)			
Month 0	5.20 ± 2.00	37.445	*<* 0.001
Month 6	3.41 ± 2.18		
Month 12	2.75 ± 2.41		
24-h urine volume (mL)			
Month 6	1117.42 ± 609.79	−2.113	0.035
Month 12	894.19 ± 591.07		
24-h ultrafiltration volume (mL)			
Month 6	601.77 ± 468.54	−1.121	0.262
Month 12	709.97 ± 600.33		
IAP (cmH_2_O)			
Month 6	16.13 ± 3.06	−2.121	0.036
Month 12	15.04 ± 2.93		

**Table 1.** Comparison of baseline characteristics and residual renal function between high-IAP and nonhigh-IAP groups.

Variables	Total (*n* = 62)	Nonhigh IAP group (*n* = 39)	High-IAP group (*n* = 23)	t/z/χ^2^	*p* Value
Age (year)	50.53 ± 13.29	50.10 ± 12.60	51.26 ± 14.64	−0.329	0.743
					
Gender, n (% female)	39(62.9)	27(69.2)	12(52.2)	1.804	0.179
BMI (kg/m^2^)	23.93 ± 3.74	23.86 ± 3.83	24.03 ± 3.68	−0.168	0.867
BSA (m^2^)	1.67 ± 0.19	1.67 ± 0.19	1.67 ± 0.18	−0.122	0.903
Blood pressure (mmHg)					
Systolic	145.08 ± 18.22	144.05 ± 20.00	146.83 ± 14.97	−0.576	0.567
Diastolic	88.16 ± 10.70	89.74 ± 10.86	85.48 ± 10.07	−1.534	0.130
HbA1c (%)	5.93 ± 1.20	5.78 ± 1.08	6.06 ± 1.35	−0.488	0.633
Heart race	77.94 ± 8.46	77.08 ± 8.56	79.39 ± 8.26	−1.189	0.234
Cause of ESKD, n (%)					
Chronic glomerulonephritis	18(29.0)	12(30.8)	6(26.1)	0.154	0.695
Diabetic nephropathy	17(27.4)	8(20.5)	9(39.1)	2.520	0.112
Hypertensive nephropathy	20(32.3)	14(35.9)	6(26.1)	0.637	0.425
Other causes	7(11.3)	5(12.8)	2(9.0)	0.006	0.936
Major comorbid conditions, n (%)					
Diabetes	3(4.8%)	1(2.5%)	2(8.6%)	0.225	0.635
Hypertension	16(25.8)	11(28.2)	5(21.7)	0.316	0.574
Coronary artery disease	9(14.5)	7(17.9)	2(9.0)	0.392	0.531
Cerebrovascular disease	8(12.9)	4(10.3)	4(17.4)	0.174	0.676
Laboratory parameters					
Hemoglobin (g/L)	105.73 ± 17.47	105.36 ± 19.27	106.35 ± 14.30	−0.214	0.832
albumin (g/L)	37.80 ± 19.86	35.64 ± 3.97	41.47 ± 32.30	−0.634	0.526
Calcium (mmol/L)	2.22 ± 0.23	2.25 ± 0.26	2.17 ± 0.16	−1.509	0.131
Phosphate (mmol/L)	1.68 ± 0.41	1.72 ± 0.46	1.60 ± 0.31	−0.649	0.517
eGFR at ESKD onset (mL/min/1.73 m^2^)	5.20 ± 2.00	4.72 ± 1.94	6.01 ± 1.88	−2.566	0.013
D/P4	0.66 ± 0.13	0.67 ± 0.13	0.65 ± 0.13	0.495	0.623
IAP (cmH_2_O)	15.53 ± 2.86	13.70 ± 1.17	18.64 ± 2.07	−10.486	<0.001
ΔUrine volume (mL)	225.32 ± 480.12	75.90 ± 314.83	478.70 ± 601.04	−3.121	0.002
ΔUltrafiltration volume (mL)	−98.68 ± 598.64	−70.72 ± 630.77	−146.09 ± 550.18	0.476	0.636
ΔResidual renal eGFR (mL/min/1.73 m^2^)	0.66 ± 1.25	0.24 ± 0.68	1.36 ± 1.64	−3.694	<0.001
ΔResidual renal eGFR (mL/min/1.73 m^2^/month)	0.11 ± 0.21	0.40 ± 0.11	0.23 ± 0.27	−3.694	<0.001

BMI: body mass index; BSA: body surface area; ESKD: end-stage kidney disease; eGFR: estimated glomerular filtration rate; D/P4: dialysate-to-plasma concentration ratio of creatinine at 4 h; IAP: intra-abdominal pressure

Δresidual renal eGFR, Δurinary volume, Δultrafiltration volume are all variables at 6 months – variables at 12 months; Δresidual renal eGFR (month) = (6-month residual kidney eGFR – 12-month residual renal eGFR)/6

### Independent risk factors associated with rapid decline in residual renal function

The study employed logistic regression analysis with sex, age, BMI, baseline eGFR, comorbidities, treatment medications during follow-up, PD prescription, and D/P4 as dependent variables. Age, IAP, BMI, baseline eGFR, and D/P4 were analyzed as continuous numerical variables, while the remaining variables were considered binary or multiclass data. Univariate analysis revealed that IAP (OR = 1.787, *p <* 0.001), diabetes status (OR = 5.314, *p <* 0.05), use of renin-angiotensin system inhibitor (RASi) medications (OR = 0.282, *p <* 0.05), and proteinuria (OR = 4.167, *p <* 0.05) were influencing factors for RRF decline. Notably, the use of RASi medications emerged as a protective factor, slowing the rapid decline in RRF. After incorporating these significant variables as confounding factors in multivariate analysis, we found that the presence of diabetes (OR = 5.275, *p <* 0.05), proteinuria (OR = 4.853, *p <* 0.05), and IAP (OR = 1.679, *p <* 0.05), remained independent risk factors for accelerated decline in RRF. Notably, for each 1 cmH_2_O increase in IAP, the risk of rapid decay in RRF increased by a factor of 1.679 (Table 3).

**Table 3.** Independent risk factors associated with rapid decline of residual.

Variables	Univariate analysis			Multivariate analysis		
	Unadjusted OR	95% CI	*p* Value	Adjusted OR	95% CI	*p* Value
Age (years)	1.016	0.977–1.057	0.424	–	–	–
Female (*versus* male)	0.682	0.241–1.933	0.471	–	–	–
Body mass index (kg/m^2^)	1.067	0.929–1.224	0.360	–	–	–
eGFR at ESKD onset (mL/min/1.73 m^2^)	1.214	0.934–1.577	0.148	–	–	–
IAP (cmH_2_0)	1.787	1.320–2.419	<0.001	1.679	1.187–2.374	0.003
Major comorbid factors				–	–	–
Diabetes mellitus (*versus* no)	5.314	1.570–17.988	0.007	5.275	1.028–27.066	0.046
Hypertension (*versus* no)	1.524	0.551–4.212	0.417	–	–	–
Treatment parameters				–	–	–
Diuretics (*versus* no)	2.644	0.930–7.518	0.068	–	–	–
RASi (*versus* no)	0.282	0.091–0.871	0.028	0.442	0.099–1.960	0.282
Proteinuria (*versus* no)	4.167	1.352–12.828	0.013	4.853	1.051–22.412	0.043
D/P4	3.727	0.069–200.849	0.518	–	–	–
PD prescription		–	–	–	–	–
1.5%*2*4 h	1.000	–	–	–	–	–
1.5%*3*4 h	1.257	0.249–6.357	0.782	–	–	–
1.5%*4*4 h	1.179	0.369–3.760	0.781	–	–	–

### Correlation analysis between IAP and the RRF decline

**Figure 2.**
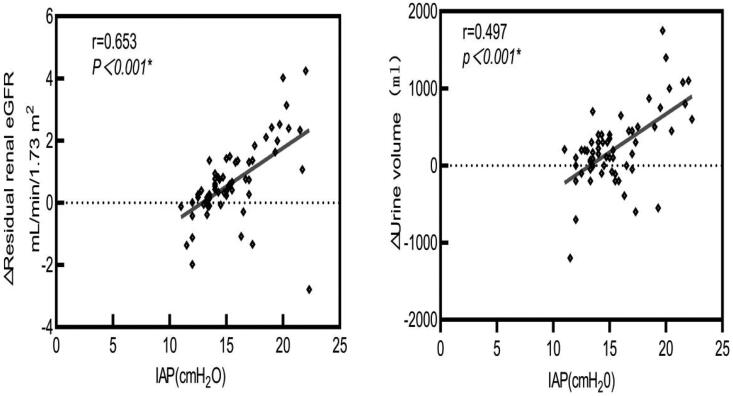
Correlation of IAP with the RRF. IAP: intra-abdominal pressure; RRF: residual renal function.

Spearman correlation analysis revealed a positive correlation between IAP and Δresidual renal eGFR (*r* = 0.653, *p* < 0.001) and Δurine volume (*r* = 0.497, *p* < 0.001). However, no significant correlation was observed between the IAP and Δultrafiltration volume (*r* = −0.056, *p* > 0.05) (Figure 2).

### Rate of RRF decline and optimal IAP threshold

**Figure 3.**
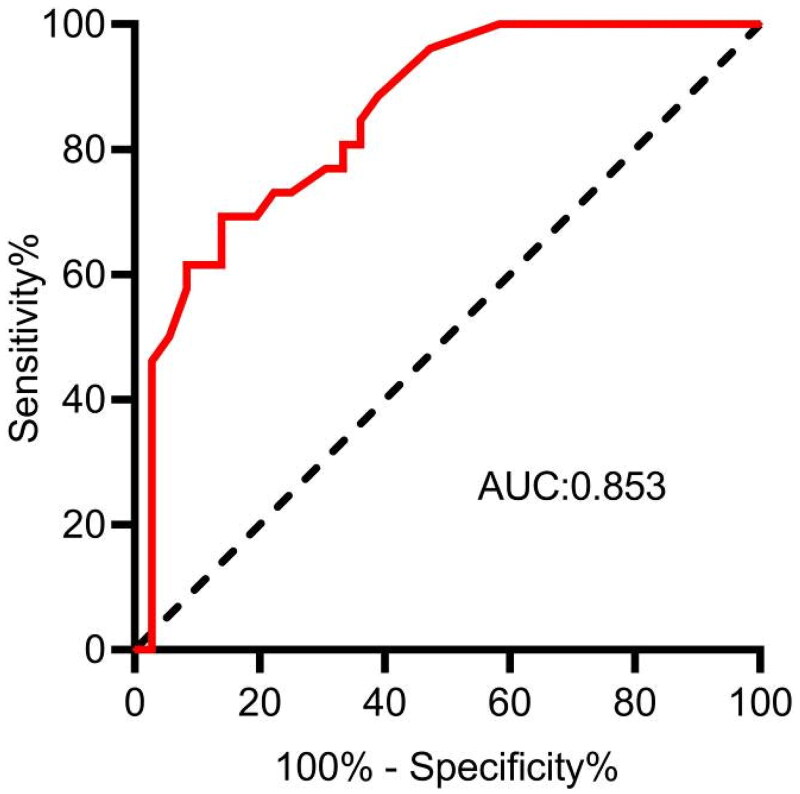
ROC curves for the IAP-predicted RRF decline. IAP: intra-abdominal pressure; RRF: residual renal function; ROC: receiver operating characteristic.

The average monthly rate of RRF decline in the 62 patients was calculated as 0.11 mL/min/1.73 m^2^. To classify RRF decline, values less than 0.11 were categorized as slow, while values greater than 0.11 were categorized as rapid. ROC curve analysis revealed that the IAP had diagnostic value for predicting the rate of RRF decline (*p <* 0.001), with an AUC of 0.853. Using the Youden index, the optimal critical value for IAP was determined to be 15.65 cmH_2_O, indicating a specificity of 86.1% and a sensitivity of 69.2% (Figure 3). Based on this threshold, 39 out of 62 patients (63%) were classified into the nonhigh IAP group (IAP < 15.65 cmH_2_O), while 23 out of 62 patients (37%) were categorized into the high IAP group (IAP ≥ 15.65 cmH_2_O).

### Comparison of patients in different IAP groups

The mean IAP for the cohort of 62 patients was 15.53 ± 2.85 cmH_2_O. In the high IAP group, the mean IAP was 18.64 ± 2.07 cmH_2_O, whereas in the nonhigh IAP group, it was 13.70 ± 1.17 cmH_2_O. Patients in the high IAP subgroup exhibited significantly greater Δresidual renal eGFR (*Z* = −3.694, *p <* 0.001), Δresidual renal eGFR (month)(*Z* = −3.694, *p <* 0.001), and Δurine volume (*Z* = −3.121, *p <* 0.01) than those in the nonhigh IAP subgroup, with a statistically significant difference between the two groups. However, there were no statistically significant differences between the two groups in terms of other indicators, such as age, BMI, BSA, or blood pressure (*p >* 0.05) (Table 2).

### Validation of the optimal critical value of 15.65 cmH_2_O

**Figure 4.**
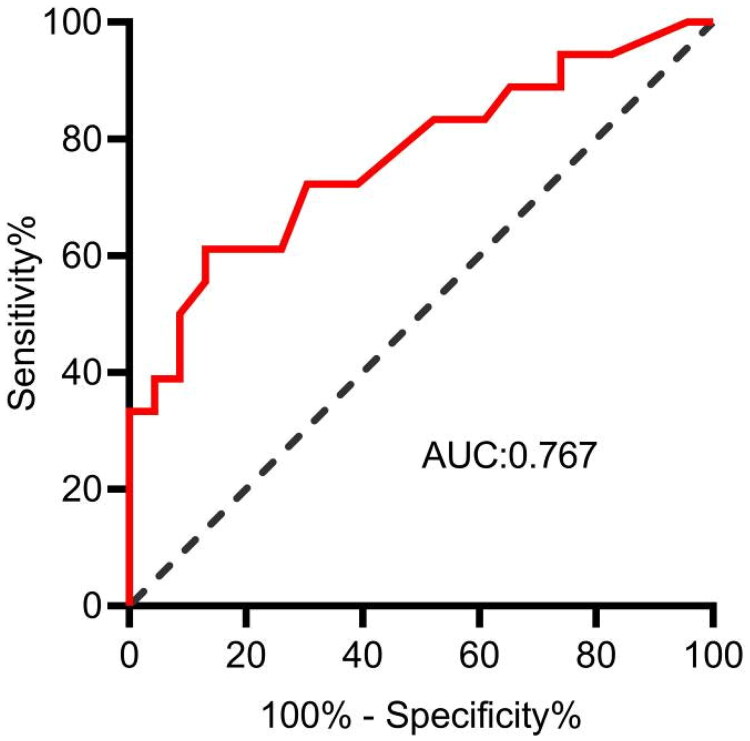
Validation threshold of 15.65 cmH_2_O.

Through a retrospective analysis of 41 additional patients with complete data at both 6 and 12 months, an AUC for the determination of the rate of RRF decline using the 15.65 cmH_2_O IAP threshold was 0.767 (95% CI [0.616, 0.918]), indicating favorable discriminative ability (*p <* 0.01) (Figure 4).

## Discussion

RRF encompasses the critical functions of filtration, reabsorption, and endocrine regulation performed by surviving renal units following renal tissue damage, and plays a pivotal role in maintaining the body’s water and electrolyte balance, regulating acid–base equilibrium, eliminating metabolic toxins, modulating calcium and phosphorus metabolism, and enhancing renal anemia management and nutritional status [8,10]. The importance of RRF has been underscored by previous studies, such as the collaborative CANUSA study conducted by Canada and the United States in 2001 [15], and the Peritoneal Dialysis Adequacy in Mexico (ADEMEX) investigation in 2002 [16]. These studies underscore the close association between RRF and the survival and quality of life of patients receiving PD.

Importantly, this study marks the inaugural inclusion of IAP as an observational parameter in the assessment of factors affecting RRF. The findings revealed a noteworthy positive correlation between IAP and alterations in RRF, signifying that heightened IAP levels are linked to an accelerated rate of RRF decline. This observation represents a novel contribution to the literature, as it has not been reported in prior studies.

The findings of our study revealed a gradual decrease in the RRF of 62 patients with PD as the treatment duration increased. Specifically, the RRF was assessed at 6 and 12 months during the stabilization phase, and the average monthly decline rate was computed at 0.11 mL/min/1.73 m^2^/month, with a median decline rate of 0.08 mL/min/1.73 m^2^/month. Notably, existing studies have reported varying results concerning the rate of RRF decline in patients with PD. For instance, a retrospective study in Taiwan involving 270 PD patients with an average follow-up of 45 months, reported a median RRF decrease rate of 0.885 mL/min/1.73 m^2^ [17]. Conversely, another study based on 130 patients with an average follow-up period of 14.4 months indicated an RRF decrease rate of 0.13 mL/min/1.73 m^2^ [18]. We employed the equation recommended by the KDIGO guidelines to calculate the RRF as the mean 24-h urinary creatinine concentration and urea clearance. Although we used the same equation as that used in the aforementioned studies, our results diverged. This discrepancy may be attributed to variations in the sample size, duration of study observation, and baseline differences in RRF between the study populations. To classify patients into either rapid or slow RRF decline categories, we established a reference point of 0.11 mL/min/1.73 m^2^ as the average rate of decline. Using ROC curve analysis, we identified an optimal IAP threshold of approximately 15.65 cmH_2_O to distinguish between rapid and slow RRF decline. This threshold reliability was subsequently confirmed in a retrospective cohort study involving 41 patients. These results reinforced the notion that maintaining the IAP below 15.65 cmH_2_O in patients with PD can effectively mitigate rapid RRF decline, thereby promoting the preservation of RRF.

Historically, several researchers have posited that clinical discomfort symptoms become notably more prevalent when the IAP exceeds 18 cmH_2_O [19–21]. Consequently, recommendations were made to maintain IAP levels below this threshold. However, our study indicates that there might be room for a more refined classification of IAP. Hence, in our study, we established the optimal cutoff value for discerning the rate of RRF decline as 15.65 cmH_2_O using ROC curve analysis. Notably, Outerelo et al. utilized a cutoff value of 13 cmH_2_O to distinguish high-risk composite outcomes in a retrospective analysis of 54 patients with PD. Their study concluded that an IAP exceeding 13 cmH_2_O was an independent predictor of patient death or transfer to HD [22]. The differences in conclusions between our study and that of Outerelo et al. may be attributed to differences in the endpoint events under investigation. Nevertheless, further exploration of the optimal IAP range is needed.

In 1947, Bradley conducted pioneering research on the impact of elevated IAP levels on human renal function. His seminal work demonstrated that increasing the IAP to 20 mmHg resulted in a substantial reduction in renal effective plasma flow (average decrease of 24.4%), GFR (average decrease of 27.5%), and urine output (average decrease of 57.4%) [23]. Similarly, an observational study involving 60 patients in the Department of Critical Care Medicine revealed a significant reduction in urine output and progressive increase in blood creatinine levels as the IAP gradually increased[24]. Further investigations using rat and porcine models subjected to varying levels of IAP revealed a negative correlation between renal blood flow and renal tissue oxygen concentration as IAP increased. Elevated IAP leads to progressive deterioration of renal function, with some animals developing anuria [4–7]. Interestingly, during laparoscopic surgery, insufflation of carbon dioxide into the abdominal cavity has the advantage of allowing visualization of the surgical procedure. However, exceeding a certain level (such as 15 mmHg) may lead to a reduction in renal blood supply, thereby causing acute kidney injury. Kopitko et al. conducted a comprehensive review on the impact of elevated IAP on the kidneys, encompassing aspects such as blood perfusion, and humoral factors, and comparing open and laparoscopic approaches, anesthesia and sedation [25,26]. Nevertheless, this perspective seems not to have garnered sufficient attention among surgeons. It is important to note that these earlier studies primarily focused on subjects with initially normal renal function, and their primary outcome was predominantly acute renal impairment. In contrast, our study focused on patients with a chronic disease course who presented with evident renal failure at the beginning of the study. Despite this distinction, our study corroborated the influence of IAP on renal function in patients undergoing PD, yielding consistent findings.

We observed changes in the IAP over time, notably, in some patients, the IAP measured at 12 months still decreased compared to that measured at 6 months, with a mean decrease of 1.1 cmH_2_O among the 62 patients at 6-month intervals. Sobrino-Pérez et al. also reported a decrease of 2.5 cmH_2_O in the IAP measured at 2-year intervals in 17 patients [27]. Durand et al. measured the IAP in patients 15 d after placement and reported that the IAP was significantly greater in the first 3 postoperative days (17 ± 3 cmH_2_O) than in the first 12 postoperative days (10 ± 4 cmH_2_O)[28]. Durand’s findings were further supported by a national study [29]. Given the differences in the selected measurement times, the results vary considerably. Comprehension of both the overall trend of the IAP and variations over different time periods, necessitates further observation over a more extended and continuous timeframe.

The presence of individual differences gives rise to notable variations in the IAP among patients with PD. These individual variations encompass sex, BMI, BSA, weight, abdominal volume, infused dialysate volume, and other factors. While some of these factors are associated with conflicting results in research, the positive correlation between BMI and IAP has garnered consistent support in the majority of clinical studies [20,30–32]. A study conducted in Italy revealed that, in comparison to BSA, BMI exhibited a stronger correlation with IAP [30]. For example, when comparing a shorter, more robust patient with a taller, leaner patient, we may find that the former exhibits a greater IAP despite having a similar BSA, owing to differences in BMI. Although this particular study did not specifically explore the relationship between BMI and IAP, it was observed that patients with elevated IAP tended to have a greater BMI than those without (23.86 ± 3.83 *vs.* 24.03 ± 3.68). Consequently, we assert that, particularly in obese patients with PD, considering BMI is imperative when formulating dialysis prescriptions. In future investigations, further research can be conducted on the factors influencing IAP. By intervening in these factors to regulate IAP, researchers can evaluate the impact of alterations in these parameters on RRF.

Several studies have underscored the importance of regular IAP measurements in PD patients [28,33,34]. It is crucial for nephrology clinicians to prioritize this assessment. Patients at risk of developing abdominal hypertension should be subjected to regular monitoring, with timely adjustments made to dialysis regimens as needed. This study has several limitations. First, the sample, which was from a single center, may not have been of sufficient size. Furthermore, the accuracy of the optimal critical value for IAP still requires further verification. Large-scale, multicenter data are needed to validate these findings. Second, considering the instability of the IAP, this study limited the measurements to two-time points, i.e. at 6 and 12 months. To obtain more accurate IAP values, it is advisable to increase the frequency of measurements, particularly for postcatheterization monitoring. Simultaneously, extending the observation period is crucial for tracking the changes in IAP and RRF over time.

## Supplementary Material

Supplemental Material
