# Supplementary material for: Intra-abdominal pressure and residual renal function decline in peritoneal dialysis: a threshold-based investigation
Source: Ren Fail. 2024 Feb 6;46(1):2312535. doi: 10.1080/0886022X.2024.2312535 (PMC10851793; doi:10.1080/0886022X.2024.2312535)
Supplement: Supplemental Material [file IRNF_A_2312535_SM6113.pdf]

Table S1 Comparison of Baseline Characteristics and RRF-Related Data Between the High-IAP and Non-High-IAP Groups of the Validation Cohort

Variables	Total （n=41）	Non-high IAP group (n=28)	High-IAP group (n=13)	t/z/χ^2	*P*-Value
Age (year)	52.34±11.63	52.46±12.92	52.08±8.69	0.098	0.922
Female (%)	18(44%)	11(39%)	7(54%)	0.764	0.382
Height (cm)	164.05±7.87	164.25±7.26	163.62±9.36	0.237	0.814
Weight (kg)	62.43±10.99	60.34±9.96	66.96±12.15	-1.836	0.074
BMI (kg/m^2)	23.34±3.72	22.32±2.96	25.54±4.33	-2.794	*0.008**
BSA (m^2)	1.76±0.16	1.74±0.15	1.81±0.18	-1.287	0.206
IAP (cmH_2O)	14.80(13.15,16.75)	13.75 (12.35,14.95)	18.30 (16.75,20.00)	-5.108	*P<0.001***
△Urine volume (ml)	220.00(107.50,550.00)	183.50(-76.00,231.25)	713.00(550.00,929.00)	-4.568	*P<0.001**
△Ultrafiltration volume (ml)	201.02±322.56	392.69±374.19	112.04±256.93	-2.807	*0.008**
△Residual kidney eGFR(mL/min/1.73 m^2)	0.68(0.35,1.28)	0.49(-0.10,0.70)	1.63(1.28,1.83)	-5.071	*P<0.001**
△Residual kidney eGFR (mL/min/1.73 m^2/Month)	0.11(0.06,0.22)	0.08(-0.02,0.12)	0.27(0.22,0.31)	-5.062	*P<0.001**
